# Dietary patterns and childhood stunting in Zimbabwe

**DOI:** 10.1186/s40795-022-00607-7

**Published:** 2022-10-12

**Authors:** Anesu Marume, Moherndran Archary, Saajida Mahomed

**Affiliations:** 1 College of Health Sciences, University of KwaZulu-Natal, Durban, South Africa; 2 Ministry of Health and Child Care, Harare, Zimbabwe; 3 King Edward VIII Hospital, Durban, South Africa

**Keywords:** Child, Dietary patterns, Maternal, Zimbabwe

## Abstract

**Background:**

Diet is one important predictor of children’s growth, and often dietary interventions can assist with reversing adverse nutrition outcomes. Traditionally research has focused on individual food items or food classes to generate an understanding of disease risk. Dietary patterns provide a holistic approach to understanding the relationship between exposure and outcome.

**Method:**

A matched case-control study was conducted. Caregivers of 450 children (225 cases, 225 controls) aged 6–59 months were asked to describe the diet their children had consumed in the previous 7 days using a Food Frequency Questionnaire. Dietary patterns were developed using factor analysis and regression analysis was conducted to assess which dietary pattern was associated with childhood stunting.

**Results:**

Three dietary patterns were identified: modern (n = 181), low animal-source (n = 158), and traditional (n = 111). Children with the low animal source dietary pattern had increased odds of being stunted (AOR 1.03, p < 0.05). Three demographic factors (Child’s age, father’s age and having a sibling < 24 months apart) were identified as significant predictors of consumption of any of the traditional and low animal source diet (P < 0.001).

**Conclusion:**

Nutrition intervention such as health education, counselling and supplementary feeding should include a holistic approach to dietary education not only focusing on promoting a balanced diet but improvement strengthening the upgrading of child’s dietary pattern taking into cognisant both quantity, and quality of nutrients provided to the child.

**Supplementary Information:**

The online version contains supplementary material available at 10.1186/s40795-022-00607-7.

## Introduction

The first 1000 days of a child starting from conception to 24 months of age is the period with the most significant child growth and development [1]. Brain development across the first 1000 days of life depends largely on the availability of both macro and micronutrients [2]. Nutritional deficiencies experienced in this period have far-reaching consequences to the child’s overall physical health. Furthermore, some micronutrients such as Vitamin B_12_, Vitamin K, iodine, zinc, iron, and folate are especially required within the first 1000 days as the growth rate of the brain size is higher thus demanding substantial energy within this period [3, 4]. Macro and micronutrient malnutrition have persisted among children below 5 years globally with developing countries mostly affected. Socioeconomic inequalities have seen millions of children globally failing to grow within the expected optimal growth due to various factors notable being limited access to adequate nutrition, adverse living conditions, high morbidity, and inadequate maternal care [5, 6].

The World Health Organization (WHO) recommends exclusive breastfeeding for the first six months of a child’s life [7]. The period in utero together with exclusive breastfeeding accounts for a significantly large proportion of a child’s first 1000 days where they rely entirely on the mother for nutrition [8, 9]. Maternal nutrition thus has a large bearing on the nutrition outcome of the child. Maternal dietary intake is linked to low birth weight and preterm deliveries [10]. Studies from Vietnam showed that a minor improvement in maternal diet before and during pregnancy was effective in increasing the mean birth weight [11, 12].

The timing, quantity, and quality of solid food provided to a growing baby may result in growth faltering [13]. A child introduced to solids may start experiencing diarrhoea and allergies. The diet a child is provided may also result in hidden hunger as the child may have micronutrient deficiency but within the normal weight to overweight range [3, 14]. Micronutrient deficiency may also exist in an undernourished child. Linear growth monitoring provides one of the most reliable ways to predict, assess and measure children’s overall wellbeing. Linear growth faltering without an appropriate diet will continue unabated beyond the first 1000 days and may have devastating effects on the growing child [1, 2]. The use of dietary patterns in assessing correlates of health outcomes provides a comprehensive consideration of individual food items, food groups, micronutrients, macronutrients, frequency of meals and quantity of food consumed within the same calculation of risk [15]. Dietary patterns vary based on various factors which include; sex, socioeconomic status, ethnic group, and culture.

Traditionally research has focused on individual food items or food classes to generate an understanding of disease risk. Dietary patterns provide a holistic approach to understanding the relationship between disease/condition with food intake, quantity, quality and nutrient interactions. Although there are numerous studies on the relationship between dietary patterns and child malnutrition, few of these have been conducted in countries with high burdens of stunting [16–20]. A majority of dietary patterns research have been conducted in eastern and western countries whose diet is different from that in Zimbabwe. Additionally, Zimbabwe like a majority of Sub-Saharan African countries follows a patriarchal system that considers male children as representatives of the head of the household, and this may result in differences in dietary consumption between boys and girls in the family [21]. Understanding such differences in infant feeding practices and the role of dietary patterns and its contribution to childhood malnutrition may provide better insight into contributory factors for stunting in such settings.

### Aim

The study objectives were to (i) identify the dietary patterns for children aged 6–59 months in Zimbabwe and (ii) ascertain the association between dietary patterns identified with childhood stunting and socio-demographic variables.

## Method

### Study design, setting and participants

A random sample of 450 caregivers-child pairs from two provinces (Manicaland and Matabeleland South Provinces) in Zimbabwe was recruited and interviewed between June and August 2021. The two provinces were purposively selected as they represent areas with the highest (Manicaland Province, 31.2%) and lowest (Matabeleland South Province, 24.2%) prevalence of stunting in the country. Participants were randomly selected to increase generalizability and representativeness. The two provinces are predominantly rural. Manicaland province is a crop and tree farming region with high rainfalls and Matabeleland South is largely an animal farming region with very low rainfalls. Children were considered eligible if they were within the ages of 6 to 59 months. The caregiver-child pair was expected to have been resident of the two selected provinces for at least 6 months prior to data collection as the study sought to assess differences between province of origin and both the child’s diet and stunting.

### Background information and covariates

The Adapted UNICEF framework for stunting was used in developing items for assessment in the study. Variables of interest for understanding children dietary patterns were categorized into three broad categories (child related factors, parental factors and household factors). Child factors considered were; child sex, age, weaning age, HIV status and anthropometric outcomes. Anthropometric outcomes were measured by trained research assistance with supervision from the principal investigator. Parental factors (both the mother and father) considered were; age, education level, and employment status. The Child Health Card and the maternal Ante-Natal Care (ANC) records were reviewed as a confirmation of responses the caregiver provided.

#### Identification of dietary patterns

Data-driven methods used to develop dietary patterns in nutritional epidemiology can be through the use of existing data collected from food frequency questionnaires (FFQ), 24-hour recall questionnaires, or dietary records [22]. Data dimensionality reduction techniques which include factor analysis, cluster analysis, and reduced rank regression (RRR), provide an approach to examining dietary patterns in relationship to health conditions and disease [15]. Factor analysis was performed as it is the more flexible method allowing for rotation of factors. An FFQ with 75 items categorized into 16 food groups was developed with guidance from the FAO guidelines for measuring household and individual dietary diversity. This FFQ was administered by trained research assistants to the 450 caregivers [23]. The questionnaire was adapted for children aged 6–59 months from a previously validated adult questionnaire [24]. Caregivers were asked how often their child was provided with each food item. Daily food consumption (energy and nutrient) was calculated by multiplying the estimated daily consumption frequency of each food item by a pre-determined nutrient content of a standard portion of the food item. To assess appropriateness of carrying out factor analysis the Kaiser-Meyer-Olkin measurement of sample adequacy (> 0.6) and Bartlett’s test of shpericity (< 0.05) were conducted. Exploratory factor analysis was conducted to the 16 food groups to identify the principal factor that accounts for the maximum fraction of the variance in the dataset. The test ensured the dietary patterns were statistically independent of one another. An assessment of the factors with the highest loading assisted in naming the dietary pattern.

### Data collection and ethical considerations

Graduate level research assistants were recruited and trained on administration of the FFQ questionnaire and assessment of child anthropometric outcome. The Ministry of Health and Child Care (MoHCC) structures gave permission to conduct the study in the two provinces. The University of KwaZulu Natal (UKZN) Biomedical Research Ethics Committee (BREC) [BE109/19] and the Medical Research Council of Zimbabwe (MRCZ) [MRCZ/A/2479] reviewed and gave ethical clearance to the study. Only mothers/caregivers who provided written informed consent were interviewed.

### Data analysis

Distribution of items from the Adapted UNICEF stunting framework and the identified dietary patterns was calculated using the Chi-squared test at p < 0.05 significance the fisher exact test was conducted for categories with less than 5 respondents. Logistic regression of childhood stunting against the dietary patterns and dietary factors was conducted at 95% confidence intervals (CIs). Any predictor with a *p*-value < 0.05 was regarded as statistically significant. The analyses were conducted using the R Statistical Computing software, 3.6.3 of the R Core Team, 2020 using the R Studio environment.

## Results

### Dietary patterns

Three dietary patterns (factors) were generated. The food items were categorized into 16 food groups (Table 1). The factors were named qualitatively by the predominant food items within the pattern. The modern dietary pattern was heavily characterized with ‘westernized’ foods most consisting of unprocessed red meats, chicken, potato chips, salads, pizza and ice cream. The low animal source dietary pattern contained a small range of food items characterized by a low consumption of meat and animal source products. The traditional dietary pattern was characterized by a high intake of fruits (mostly wild fruits), meat, milk, milk products, insects and worms. Some of the food items within the traditional dietary pattern were seasonal. A heavy starch diet was observed across all three classes. Caregivers provided their children with a maize meal in form of porridge or sadza (pap/thick porridge), and reported to add multiple other food items to the porridge fed. The most commonly added food items to the porridge were cooking oil (73%), peanut butter (68%), milk (47%), and fruits (33%).

**Table 1:** Food items consumed by children 6–59 months, Zimbabwe

Food groups	Factor 1 (Modern dietary pattern)	Factor 2 (Low animal source)	Factor 3 (Traditional)	Communality
1. Maize	0.40	**0.43**	0.39	0.82
2. Cereals	**0.51**	0.20	-0.17	0.32
3. White roots and tubers	0.18	0.26	**0.31**	0.20
4. Organ meats	**0.39**	0.21	0.26	0.28
5. Fish	0.26	0.14	**0.49**	0.40
6. Flesh meats	**0.60**	0.36	0.14	0.30
7. Eggs	**0.48**	0.15	0.28	0.36
8. Milk and milk products	0.32	0.08	**0.55**	0.29
9. Insects and worms	0.25	0.16	**0.62**	0.49
10. Vitamin A rich fruits and veg	0.19	0.38	**0.73**	0.55
11. Legumes, nuts and seeds	0.22	**0.57**	0.25	0.35
12. Beverages	**0.81**	0.13	0.18	0.21
13. Dark green leafy vegetables	0.07	0.33	**0.89**	0.30
14. Fruits	0.36	**0.55**	0.17	0.25
15. Deserts	**0.40**	-0.11	-0.05	0.30
16. Oils and fats	**0.83**	0.26	0.14	0.51
Total communalities				5.93
Cronbach’s alpha	0.81	0.77	0.69	

**Fig. 1:**
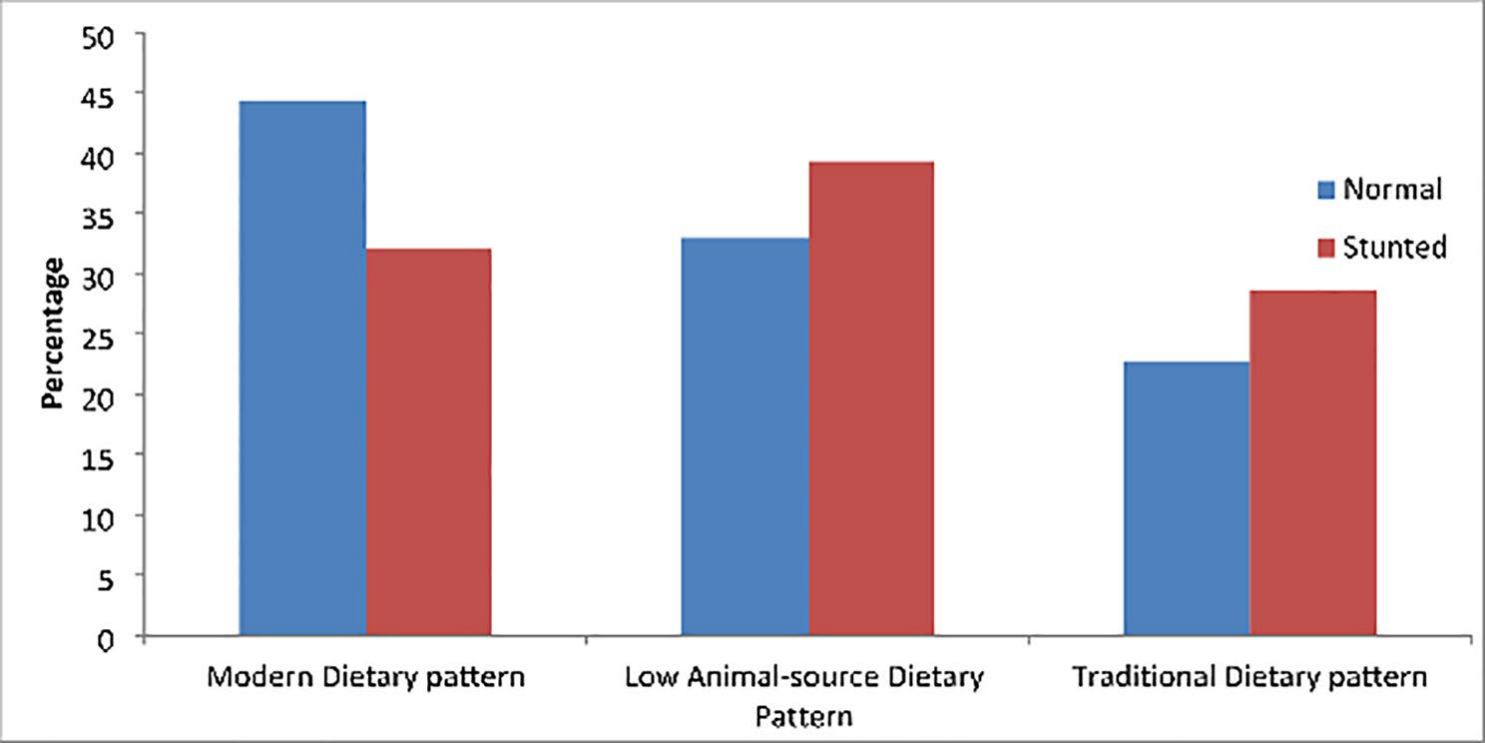
Child dietary patterns among Zimbabwean children, 2021

The modern dietary pattern (n = 181, 40%) was the most common followed by the low animal source dietary pattern (n = 158, 35%), and the traditional dietary pattern (n = 111, 25%). There was a significant difference between the dietary patterns by child stunting (p = 0.016). (Fig. 1)

### Respondents’ characteristics and dietary patterns

Childhood, parental and household factors were compared to the three dietary patterns (Table 2). The mean age of children with the traditional dietary pattern was significantly higher in comparison with children with other dietary patterns. Children of younger mothers and fathers (mean age 23 years and 29.1 years respectively) were more likely to have the modern dietary pattern, and children of older mothers and fathers (mean age 30.8 years and 35.2 years respectively) were more likely to have the low animal source dietary pattern. Children in larger households were more likely to consume the modern dietary pattern in comparison to households with four and fewer people while children in larger households were more likely to consume low animal-source dietary patterns. There was no observed difference in the dietary patterns by maternal and paternal education or occupation.

**Table 2:** Respondents characteristics, dietary patterns, and child stunting, Zimbabwe

Variables		Modern (N = 181)	Low animal source (N = 158)	Traditional (N = 111)	P-value
					0.012
Province	Manicaland	88 (48.6%)	84 (53.2%)	53 (47.7%)	
	Matabeleland south	93 (51.4%)	74 (46.8%)	58 (52.3%)	
**Child factors**					0.036
Child’s Sex	Female	95 (52.5%)	65 (41.1%)	45 (40.5%)	
	Male	86 (47.5%)	73 (46.2%)	66 (59.5%)	< 0.001
Child’s Age (months)	Mean ± SD	16.1 ± 11.2	15.8 ± 11.5	23.3 ± 15.3	0.015
Age weaning	< 24 months	79 (43.6%)	75 (47.5%)	66 (59.5%)	
	≥ 24 months	102 (56.4%)	83 (52.5%)	45 (40.5%)	0.024
Height-for-age	Normal	133 (73.5%)	99 (62.7%)	68 (61.3%)	
	Stunted	48 (26.5%)	59 (37.3%)	43 (38.7%)	0.345
Weight-for age	Underweight	55 (30.4%)	35 (22.2%)	33 (29.7%)	0.089
	Overweight	30 (16.6%)	35 (22.2%)	24 (21.6%)	< 0.001
MUAC	Normal	171 (94.5%)	135 (85.4%)	104 (93.7%)	
	Wasted	10 (5.5%)	24 (15.2%)	7 (6.3%)	0.645
Child HIV status	Negative	172 (95.0%)	149 (94.3%)	106 (95.5%)	
	Positive	9 (5.0%)	9 (5.7%)	5 (4.5%)	
**Parental factors**					< 0.001
Maternal age (years)	Mean ± SD	23.0 ± 3.83	30.8 ± 8.95	27.7 ± 6.20	< 0.001
Paternal age (years)	Mean ± SD	29.1 ± 3.28	35.2 ± 11.4	32.4 ± 6.39	0.033
Parental survival	Both alive	149 (82.3%)	133 (84.2%)	94 (84.7%)	
	Both dead	17 (9.4%)	16 (10.1%)	13 (11.7%)	
	Father alive	5 (2.8%)	1 (5.6%)	1 (0.9%)	
	Mother alive	10 (5.5%)	8 (5.7%)	3 (2.7%)	0.287
Maternal education	No School	4 (2.2%)	7 (5.0%)	5 (4.5%)	
	School	177 (97.8%)	151 (95.6%)	106 (95.5%)	0.887
Paternal education	No education	6 (3.3%)	7 (5.0%)	5 (4.5%)	
Maternal occupation	School	175 (96.7%)	151 (95.6%)	106 (95.5%)	0.369
	Formal	25 (13.8%)	23 (14.6%)	10 (9.0%)	
	Informal	47 (26.0%)	46 (29.1%)	37 (33.3%)	
	Unemployed	109 (60.2%)	90 (57.0%)	64 (57.7%)	0.487
Paternal occupation	Formal	60 (33.1%)	61 (38.6%)	43 (38.7%)	
	Informal	51 (28.2%)	40 (25.3%)	29 (26.1%)	
	Unemployed	70 (38.7%)	57 (36.1%)	39 (35.1%)	0.007
Maternal HIV status	Negative	135 (74.6%)	116 (73.4%)	98 (88.3%)	
	Positive	46 (25.4%)	42 (26.6%)	13 (11.7%)	
**Household factors**					< 0.001
Number in household	< 4 people	57 (31.5%)	51 (32.3%)	33 (29.7%)	
	≥ 4 people	124 (68.5%)	107 (67.7%)	78 (70.3%)	< 0.001
Number of siblings	Multiple	137 (75.7%)	103 (65.2%)	84 (75.7%)	
	Only child	44 (24.3%)	55 (34.8%)	27 (24.3%)	0.658
Child spacing	< 24 months apart	84 (46.4%)	80 (50.6%	63 (56.8%)	
	≥ 24 months apart	97 (53.6%)	78 (49.4%	48 (43.2%)	< 0.001
Religion	Christianity	146 (80.7%)	132 (83.5%)	95 (85.6%)	
	Other	35 (19.3%)	26 (16.5%)	16 (14.4%)	0.468
Ever breastfed	Yes	172 (95.0%)	149 (94.3%)	107 (96.4%)	
	No	9 (5.0%)	9 (6.4%)	4 (3.6%)	< 0.001
Water source	Protected	144 (79.6%)	134 (84.8%)	28 (25.2%)	
	Not protected	37 (20.4%)	24 (17.1%)	83 (74.8%)	0.323
Wealth index	High	15 (8.3%)	24 (15.2%)	16 (14.4%)	
	Middle	86 (47.5%)	70 (44.3%	48 (43.2%)	

*Significance testing: Chi-squared analysis (n > 5), Fisher’s exact (n < 5)

### Factors associated with childhood dietary patterns

Table 3 summarizes the factors associated with childhood dietary patterns. Children who were classified as consuming the low animal source dietary pattern had a 1.47 times increased likelihood to be underweight compared to children with normal weight. Children consuming the low animal source dietary pattern had increased odds of having multiple diarrhoeal (AOR 1.10) and respiratory infections (AOR 1.11). The employment status of the father, the wealth level of the household, child spacing and number of siblings a child had were all found to be significantly related with the dietary pattern a child was exposed to (Table 3).

**Table 3:** Factors associated with childhood dietary patterns in Zimbabwe

	Modern dietary pattern	Traditional	Low animal source
		**AOR(65%CI, p-value)**	**AOR(65%CI, p-value)**
Matabeleland South	Reference	0.84(0.47–1.60, p = 0.247)	0.13(0.10–0.61, p = 0.003)
**Child factors**			
Child mean age	Reference	1.18(1.12–1.37, p < 0.001)	1.83(1.50–1.01, p < 0.001)
Child sex (Male)	Reference	1.36(0.61–3.18, p = 0.336)	1.10(0.35–1.83, p = 0.831)
Stunted height	Reference	0.66(0.21–1.07, p = 0.061)	1.03(1.00-1.08, p = 0.051)
Overweight	Reference	0.51(0.18–1.56, p = 0.150)	0.55(0.18–1.64, p = 0.160)
Underweight	Reference	0.66(0.13–1.87, p = 0.316)	1.47(1.06–1.62, p = 0.008)
Wasted	Reference	0.53(0.06–3.13, p = 0.388)	1.68(0.18–10.58, p = 0.583)
**Disease and infections (past two weeks)**			
HIV positive (child)	Reference	1.36(0.58–3.64, p = 0.310)	0.81(0.31–1.16, p = 0.628)
Diarrhoea	Reference	1.31(0.63–8.36, p = 0.166)	3.16(0.78–11.46, p = 0.105)
Multiple Diarrhoea Episodes	Reference	0.56(0.13–1.18, p = 0.311)	1.10(1.05–5.86, p = 0.035)
Respiratory infection	Reference	0.11(0.03–0.34, p < 0.001)	1.11(1.06–3.83, p = 0.013)
Fever	Reference	8.30(1.61–10.61, p < 0.001)	0.88(0.18–1.81, p = 0.831)
**Parental factors**			
Mean Age Father	Reference	1.61(1.38–1.65, p < 0.001)	1.03(1.81–1.33, p < 0.001)
Mean Age Mother	Reference	1.30(0.11–1.87, p = 0.111)	1.38(0.67–1.85, p = 0.061)
Father Informally employed	Reference	0.85(0.30–1.34, p = 0.863)	0.83(0.15–1.80, p = 0.858)
Father Unemployed	Reference	1.11(1.08–10.68, p = 0.006)	1.16(1.08–4.86, p = 0.031)
Mother Informally employed	Reference	1.83(0.38–6.13, p = 0.360)	1.53(0.13–6.86, p = 0.653)
Mother Unemployed	Reference	1.60(0.34–8.36, p = 0.538)	3.31(0.53–10.61, p = 0.101)
**Household factors**			
Non-Christian religion	Reference	0.81(0.18–1.53, p = 0.833)	0.38(0.13–1.81, p = 0.158)
More than 3 in Household	Reference	1.33(0.87–6.53, p = 0.105)	13.01(3.16–36.81,p < 0.001)
More than 1 siblings	Reference	0.56(0.17–1.81, p = 0.335)	1.33(0.36–3.50, p = 0.535)
Child spacing (< 13 months)	Reference	1.03(1.02–1.53, p < 0.001)	1.80(1.15-3.00, p < 0.001)
Unprotected water source	Reference	0.16(0.06–1.81, p = 0.066)	0.51(0.16–1.61, p = 0.136)
No Farming Land	Reference	0.35(0.18–1.16, p = 0.068)	1.16(1.10–5.81, p = 0.018)
Low wealth household	Reference	1.01(1.06–1.33, p = 0.055)	0.81(0.13–3.85, p = 0.688)
Middle wealth household	Reference	1.18(1.03–1.86, p = 0.010)	0.31(0.06–1.81, p = 0.181)

### Child feeding, dietary patterns, and childhood stunting

There was no significant association between the traditional dietary pattern and childhood stunting. Children within the low animal source dietary pattern were 2.22 times more likely to be stunted (p = 0.05) (Table 3). Children who were fed milk replacements such as porridge and/or formula milk at birth were 3 times more likely to be stunted (p = 0.047). Children who had never been breastfed were 4 times more likely to be stunted (p < 0.05). Nearly half (46%) of the children had complementary feed introduced before the age of 6 months and were 3 times more likely to be stunted (p = 0.013). A majority of children (53%) had stopped breastfeeding before the age of 24 months and were 2 times more likely to be stunted (p = 0.014). Forty-five (11%) mothers reported consuming fewer meals during pregnancy and lactation (n = 14, 3%) (Table 4). However, a larger proportion stated that they consumed an extra meal during pregnancy (n = 256, 69%) and lactation (n = 118, 32%). Children from mothers who reported that the meals they consumed remained constant when they were pregnant, or were reduced were more likely to be stunted OR 1.84(p = 0.01) and 2.29 respectively (p = 0.03)) Similarly, a reduction in the number of meals mothers consumed while lactating had an increased likelihood of children becoming stunted (p < 0.05) The food variety mothers consumed during pregnancy and lactation had no significant relationship with childhood stunting.

**Table 4:** Maternal and child feeding, dietary patterns, and childhood stunting

Variable	Explanatory	Normal Height (N = 300)	Stunted growth (N = 150)	Adjusted OR (95% CI, p-value)
**Child dietary factors**				
Dietary pattern	Modern	133 (44.3%)	48 (32.0%)	Reference
	Low animal source	99 (28.0%)	59 (37.3%)	2.22 (1.87–3.31, p = 0.002)
	Traditional	68 (22.7%)	43 (28.7%)	0.69 (0.01–6.48, p = 0.298)
Feed on delivery	Milk replacement	8 (2.7%)	9 (6.0%)	2.98 (1.02–9.21, p = 0.047)
	Breast milk	292 (97.3%)	141 (94.0%)	Reference
History of breastfeeding	Ever	293 (97.7%)	137 (91.3%)	Reference
	Never	7 (2.3%)	13 (8.7%)	3.16 (2.14–9.59, p = 0.009)
Meals per day	< 3 meals	195 (65.0%)	89 (59.3%)	0.72 (0.39–1.30, p = 0.274)
	≥ 3 meals	105 (35.0%)	61 (40.7%)	Reference
Breastfeeding cessation	< 24 months	151 (50.3%)	88 (58.7%	1.91 (1.84–5.98, p = 0.014)
	≥ 24 months	149 (49.7%)	62 (41.3%)	Reference
Age introduced complementary feed	< 6 months	160 (53.3%)	83 (55.3%)	3.06 (1.28–7.51, p = 0.013)
	≥ 6 months	140 (46.7%)	67 (44.7%)	Reference
**Maternal food consumption**				
*Pregnancy*				
Number of meals	Increased	162 (61.7%)	63 (59.3%)	Reference
	No change	71 (27.0%)	34 (31.3%)	1.84 (1.24–4.10, p = 0.010)
	Reduced	29 (11.3%)	10 (9.3%)	2.29 (1.48–3.48, p = 0.026)
Food variety	Improved	109 (41.7%)	45 (42.0%)	Reference
	No change	153 (58.3%)	62 (58.0%)	0.67 (0.30–1.47, p = 0.321)
*Lactation*				
Number of meals	Increased	78 (29.7%)	26 (24.0%)	Reference
	No change	179 (68.3%)	74 (69.3%)	0.55 (0.21–1.43, p = 0.213)
	Reduced	5 (2%)	7 (6.7%)	1.76 (1.02–3.12, p = 0.047)
Food variety	Improved	24 (9.3%)	13 (12.0%)	Reference
	No Change	238 (90.7%)	94 (88.0%)	0.98 (0.65–1.48, p = 0.922)

## Discussion

This study reports on three distinct dietary patterns and their relationship with childhood, parental and household factors in Zimbabwe. Dietary patterns were differentiated from one another qualitatively both by the degree of their overall diversity and by specific food items consumed. We found differences in the dietary pattern based on the child’s gender. Girls were more likely to have the low animal source diet while boys were more likely have the low animal source dietary pattern. Similar findings were noted in a study conducted in Bangladesh which found significant difference between dietary patterns with gender, socio-economic status and childhood stunting [20]. A study in China argued that the difference in diet by gender were due to the different social values and roles communities place on children by gender [25].

Due to the chronic nature of stunting, valid associations are more likely to be produced when overall dietary patterns are considered in contrast to an assessment by specific food items [16]. While classification of dietary patterns differs between these studies, there is a general agreement that a diet with the highest diversity of food groups was protective of under-nutrition [17, 18, 26, 27], similar to our findings.

More often the modern dietary pattern is a result of nutrition transition from the traditional diet to a more global/western dietary pattern [28]. Most studies identify the modern dietary pattern within populations who fall under the high economic class of the society [20, 29]. Similar to other studies, the modern dietary pattern in this study was characterized by an assortment of food items such as pizza, ice cream and fast foods that may as well fit within the western diet classification as defined by a majority of studies. A study in Kenya found that children with a dietary pattern that included locally available foods were less likely to be stunted compared to children with a dietary pattern defined as western diet. This finding is in contrast to a study of children in the East Mediterranean that found children with a western diet were less likely to be stunted compared to a diet of locally available foods [19, 30].

The traditional dietary pattern is characterized by consumption of insects, worms, seeds, legumes and vegetables which are seasonal but mostly freely accessible in the environment [31–34]. In the current study, we found no association between the traditional dietary pattern and childhood stunting. A study conducted in Iran which found similar findings concluded that due to the seasonality of traditional food items, children who consumed this dietary pattern were more likely to be exposed to both a balanced and an unbalanced diet [16]. In contrast to our findings, the study in Kenya reported that the indigenous diet termed ‘traditional dietary pattern’ is more likely to be protective of adverse nutrition outcomes as it more often contains a wide variety of food items that are easily accessible to most ordinary community members (Tanaka et al., 2019). Multiple studies have confirmed the role of dietary patterns with nearly all forms of childhood malnutrition [35–37]. Childhood illnesses such as fever, worms and diarrhoea were found to significant predictors of childhood stunting. Children who are ill are more likely to report high levels of loss of appetite and selective consumption of food [38].

Multiple studies have been conducted globally that assessed the differences in dietary pattern by the socio-economic status of households. A study conducted in 12 countries globally found significant differences in child dietary pattern by household economic status [39]. Household wealth in our study was significant determinant of the diet children consumed with children within both the low wealth index and the middle wealth index significantly more likely to follow the traditional dietary pattern in comparison with those within the high wealth index. Other factors that can be used as proxies for household wealth such as paternal employment and ownership of farming land were also identified as significant determinants of the dietary pattern a child was exposed to.

### Limitations

Maternal diet is an important predictor of childhood stunting and however this variable has a high probability of recall bias as the period between exposure and outcome would be nearly 5 years for some mothers. Similarly, age of weaning and time of commencement of complementary feed may have also been affected by recall bias. Additionally collecting a 7-day diet may not factor in seasonal changes and other changes of importance that can help determine access of the food items by children hence this may not accurately reflect the diet children consume throughout the year. These limitations can be addressed by a longitudinal study. Future studies can also assess the role of confounding and effect modification in the relationship between dietary patterns and childhood stunting by variables such as province, household wealth, child’s age among others within the stunting framework.

## Conclusion

This study confirmed presence of a significant relationship between child dietary patterns and stunting. Interventions such as supplementary feeding programmes, nutrition counselling and income generating projects which can result in improved dietary diversity at household should be promoted.

## Electronic supplementary material

Below is the link to the electronic supplementary material.

Supplementary Material 1

Supplementary Material 2

## Data Availability

The dataset generated and analysed during the current study are mentioned in supplementary files.

## Declarations

### Author details

^1^College of Health Sciences, University of KwaZulu-Natal, South Africa.

^2^Ministry of Health and Child Care, Zimbabwe.

^3^King Edward VIII Hospital, Durban, South Africa.

## Abbreviations

FFQ: Food frequency Questionnaire.
NNS: National Nutrition Survey.
ZDHS: Zimbabwe Demographics Health Survey.
