# Supplementary material for: Dietary patterns and childhood stunting in Zimbabwe
Source: BMC Nutr. 2022 Oct 12;8:111. doi: 10.1186/s40795-022-00607-7 (PMC9555084; doi:10.1186/s40795-022-00607-7)
Supplement: Supplementary file 1 — Supplementary Material 1 [file 40795_2022_607_MOESM1_ESM.docx]

Supplementary Table 1: Dietary patterns by stunting diagnosis

|  | **Normal Height Children** | | | | | **Stunted Height Children** | | | | |
| --- | --- | --- | --- | --- | --- | --- | --- | --- | --- | --- |
| **Dietary patterns** | **Least Diverse (N=84)** | **Low animal source (N=15)** | **Most Diverse (N=133)** | **Traditional (N=68)** | **p-value** | **Least Diverse (N=56)** | **Low animal source (N=3)** | **Most Diverse (N=48)** | **Traditional (N=43)** | **p-value** |
| **Province** |  |  |  |  | 0.121 |  |  |  |  | 0.014 |
| Manicaland | 43 (51.2%) | 3 (20.0%) | 70 (52.6%) | 34 (50.0%) |  | 37 (66.1%) | 1 (33.3%) | 18 (37.5%) | 19 (44.2%) |  |
| Matabeleland South | 41 (48.8%) | 12 (80.0%) | 63 (47.4%) | 34 (50.0%) |  | 19 (33.9%) | 2 (66.7%) | 30 (62.5%) | 24 (55.8%) |  |
| **Age (months)** |  |  |  |  | 0.173 |  |  |  |  | <0.001 |
| Mean ±SD (CV %) | 19.3±12.6(65.5) | 12.0±4.44(37.0) | 18.3±11.7(63.9) | 22.9±17.1(74.8) |  | 10.4±6.78(64.9) | 13.0±0(0) | 9.88±6.40(64.8) | 23.9±12.2(51.1) |  |
| Median(Q1-Q3) | 15.0(9.00-27.0) | 9.00(9.00-14.0) | 13.0(9.00-23.0) | 16.0(9.00-39.3) |  | 9.00(8.00-10.0) | 13.0(13.0-13.0) | 9.00(8.00-10.0) | 20.0(16.0-26.5) |  |
| Min-Max | 6.00-46.0 | 8.00-22.0 | 6.00-52.0 | 6.00-59.0 |  | 6.00-55.0 | 13.0-13.0 | 6.00-52.0 | 7.00-52.0 |  |
| **Sex** |  |  |  |  | 0.007 |  |  |  |  | 0.014 |
| Female | 24 (28.6%) | 8 (53.3%) | 69 (51.9%) | 32 (47.1%) |  | 30 (53.6%) | 3 (100%) | 26 (54.2%) | 13 (30.2%) |  |
| Male | 60 (71.4%) | 7 (46.7%) | 64 (48.1%) | 36 (52.9%) |  | 26 (46.4%) | 0 (0%) | 22 (45.8%) | 30 (69.8%) |  |
| **Number in household** |  |  |  |  | <0.001 |  |  |  |  | 0.163 |
| Less than 4 people | 18 (21.4%) | 15 (100%) | 33 (24.8%) | 16 (23.5%) |  | 18 (32.1%) | 0 (0%) | 24 (50.0%) | 17 (39.5%) |  |
| More than 4 people | 66 (78.6%) | 0 (0%) | 100 (75.2%) | 52 (76.5%) |  | 38 (67.9%) | 3 (100%) | 24 (50.0%) | 26 (60.5%) |  |
| **Parental survival** |  |  |  |  | 0.774 |  |  |  |  | <0.001 |
| Both alive | 68 (81.0%) | 12 (80.0%) | 107 (80.5%) | 54 (79.4%) |  | 53 (94.6%) | 0 (0%) | 42 (87.5%) | 40 (93.0%) |  |
| Both dead | 10 (11.9%) | 3 (20.0%) | 13 (9.8%) | 10 (14.7%) |  | 1 (1.8%) | 2 (66.7%) | 4 (8.3%) | 3 (7.0%) |  |
| Father alive | 0 (0%) | 0 (0%) | 4 (3.0%) | 1 (1.5%) |  | 0 (0%) | 1 (33.3%) | 1 (2.1%) | 0 (0%) |  |
| Mother alive | 6 (7.1%) | 0 (0%) | 9 (6.8%) | 3 (4.4%) |  | 2 (3.6%) | 0 (0%) | 1 (2.1%) | 0 (0%) |  |
| **Child HIV status** |  |  |  |  | 0.696 |  |  |  |  | 0.619 |
| Negative | 79 (94.0%) | 14 (93.3%) | 125 (94.0%) | 66 (97.1%) |  | 53 (94.6%) | 3 (100%) | 47 (97.9%) | 40 (93.0%) |  |
| Positive | 5 (6.0%) | 1 (6.7%) | 8 (6.0%) | 2 (2.9%) |  | 3 (5.4%) | 0 (0%) | 1 (2.1%) | 3 (7.0%) |  |
| **Wasting** |  |  |  |  | <0.001 |  |  |  |  | 0.804 |
| Normal | 80 (95.2%) | 7 (46.7%) | 129 (97.0%) | 68 (100%) |  | 45 (80.4%) | 3 (100%) | 42 (87.5%) | 36 (83.7%) |  |
| Wasted | 4 (4.8%) | 8 (53.3%) | 4 (3.0%) | 0 (0%) |  | 11 (19.6%) | 0 (0%) | 6 (12.5%) | 7 (16.3%) |  |
| **Weight for age** |  |  |  |  |  |  |  |  |  |  |
| Underweight | 27 (32.1%) | 6 (40.0%) | 43 (32.3%) | 26 (38.2%) | 0.782 | 2 (3.6%) | 0 (0%) | 12 (25.0%) | 7 (16.3%) | 0.011 |
| Overweight | 16 (19.0%) | 3 (20.0%) | 15 (11.3%) | 17 (25.0%) | 0.089 | 16 (28.6%) | 0 (0%) | 15 (31.2%) | 7 (16.3%) | 0.287 |
| **Maternal age** |  |  |  |  | <0.001 |  |  |  |  | <0.001 |
| Mean ±SD (CV %) | 32.7±9.45(28.9) | 28.7±4.80(16.7) | 23.4±4.24(18.1) | 29.5±6.39(21.7) |  | 27.8±7.31(26.2) | 27.7±3.79(13.7) | 21.9±1.96(8.9) | 24.8±4.66(18.8) |  |
| Median(Q1-Q3) | 35.0(30.0-40.0) | 30.0(30.0-31.0) | 25.0(20.0-26.0) | 32.0(27.0-33.0) |  | 26.0(21.8-32.3) | 26.0(25.5-29.0) | 22.0(22.0-23.0) | 24.0(24.0-25.0) |  |
| Min-Max | 16.0-51.0 | 16.0-31.0 | 15.0-42.0 | 17.0-43.0 |  | 19.0-48.0 | 25.0-32.0 | 16.0-24.0 | 20.0-47.0 |  |
| **Maternal education** |  |  |  |  | 0.562 |  |  |  |  | 0.058 |
| No School | 1 (1.2%) | 0 (0%) | 4 (3.0%) | 0 (0%) |  | 6 (10.7%) | 0 (0%) | 0 (0%) | 5 (11.6%) |  |
| Attended School | 83 (98.8%) | 15 (100%) | 129 (97.0%) | 68 (100%) |  | 50 (89.3%) | 3 (100%) | 48 (100%) | 38 (88.4%) |  |
| **Maternal occupation** |  |  |  |  | 0.394 |  |  |  |  | 0.720 |
| Formal | 20 (23.8%) | 1 (6.7%) | 25 (18.8%) | 10 (14.7%) |  | 1 (1.8%) | 0 (0%) | 0 (0%) | 0 (0%) |  |
| Informal | 11 (13.1%) | 4 (26.7%) | 26 (19.5%) | 17 (25.0%) |  | 30 (53.6%) | 1 (33.3%) | 21 (43.8%) | 20 (46.5%) |  |
| Unemployed | 53 (63.1%) | 10 (66.7%) | 82 (61.7%) | 41 (60.3%) |  | 25 (44.6%) | 2 (66.7%) | 27 (56.2%) | 23 (53.5%) |  |
| **Maternal HIV status** |  |  |  |  | 0.016 |  |  |  |  | 0.072 |
| Negative | 67 (79.8%) | 8 (53.3%) | 100 (75.2%) | 60 (88.2%) |  | 38 (67.9%) | 3 (100%) | 35 (72.9%) | 38 (88.4%) |  |
| Positive | 17 (20.2%) | 7 (46.7%) | 33 (24.8%) | 8 (11.8%) |  | 18 (32.1%) | 0 (0%) | 13 (27.1%) | 5 (11.6%) |  |
| **Paternal age** |  |  |  |  | <0.001 |  |  |  |  | <0.001 |
| Mean ±SD (CV %) | 43.8±5.07(11.6) | 48.1±4.95(10.3) | 29.7±3.60(12.1) | 36.8±2.19(6.0) |  | 22.3±2.38(10.7) | 21.0±0(0) | 27.4±0.962(3.5) | 25.5±4.40(17.3) |  |
| Median(Q1-Q3) | 43.0(40.0-47.0) | 49.0(49.0-49.0) | 31.0(27.0-33.0) | 37.0(35.0-38.3) |  | 22.5(20.8-24.0) | 21.0(21.0-21.0) | 28.0(27.0-28.0) | 29.0(21.0-29.0) |  |
| Min-Max | 18.0-58.0 | 36.0-54.0 | 18.0-34.0 | 32.0-45.0 |  | 18.0-29.0 | 21.0-21.0 | 26.0-29.0 | 18.0-30.0 |  |
| **Paternal occupation** |  |  |  |  | 0.140 |  |  |  |  | 0.004 |
| Formal | 29 (34.5%) | 9 (60.0%) | 55 (41.4%) | 35 (51.5%) |  | 23 (41.1%) | 0 (0%) | 5 (10.4%) | 8 (18.6%) |  |
| Informal | 20 (23.8%) | 4 (26.7%) | 38 (28.6%) | 13 (19.1%) |  | 15 (26.8%) | 1 (33.3%) | 13 (27.1%) | 16 (37.2%) |  |
| Unemployed | 35 (41.7%) | 2 (13.3%) | 40 (30.1%) | 20 (29.4%) |  | 18 (32.1%) | 2 (66.7%) | 30 (62.5%) | 19 (44.2%) |  |
| **Paternal education** |  |  |  |  | 0.295 |  |  |  |  | 0.065 |
| No school | 2 (2.4%) | 0 (0%) | 6 (4.5%) | 0 (0%) |  | 5 (8.9%) | 0 (0%) | 0 (0%) | 5 (11.6%) |  |
| Attended school | 82 (97.6%) | 15 (100%) | 127 (95.5%) | 68 (100%) |  | 51 (91.1%) | 3 (100%) | 48 (100%) | 38 (88.4%) |  |
| **Religion** |  |  |  |  | <0.001 |  |  |  |  | 0.462 |
| Christianity | 76 (90.5%) | 5 (33.3%) | 109 (82.0%) | 57 (83.8%) |  | 48 (85.7%) | 3 (100%) | 37 (77.1%) | 38 (88.4%) |  |
| Other | 8 (9.5%) | 10 (66.7%) | 24 (18.0%) | 11 (16.2%) |  | 8 (14.3%) | 0 (0%) | 11 (22.9%) | 5 (11.6%) |  |
| **Number siblings** |  |  |  |  | <0.001 |  |  |  |  | 0.324 |
| Multiple | 55 (65.5%) | 1 (6.7%) | 103 (77.4%) | 47 (69.1%) |  | 44 (78.6%) | 3 (100%) | 34 (70.8%) | 37 (86.0%) |  |
| Only child | 29 (34.5%) | 14 (93.3%) | 30 (22.6%) | 21 (30.9%) |  | 12 (21.4%) | 0 (0%) | 14 (29.2%) | 6 (14.0%) |  |
| **Ever breastfed** |  |  |  |  | 1.000 |  |  |  |  | 0.680 |
| No | 3 (3.6%) | 0 (0%) | 4 (3.0%) | 2 (2.9%) |  | 6 (10.7%) | 0 (0%) | 5 (10.4%) | 2 (4.7%) |  |
| Yes | 81 (96.4%) | 15 (100%) | 129 (97.0%) | 66 (97.1%) |  | 50 (89.3%) | 3 (100%) | 43 (89.6%) | 41 (95.3%) |  |
| **Water source** |  |  |  |  | <0.001 |  |  |  |  | <0.001 |
| Not protected | 0 (0%) | 0 (0%) | 34 (25.6%) | 49 (72.1%) |  | 24 (42.9%) | 0 (0%) | 3 (6.2%) | 34 (79.1%) |  |
| Protected | 84 (100%) | 15 (100%) | 99 (74.4%) | 19 (27.9%) |  | 32 (57.1%) | 3 (100%) | 45 (93.8%) | 9 (20.9%) |  |
| **Child spacing** |  |  |  |  | **0.220** |  |  |  |  | **0.835** |
| Before 24 months | 49 (58.3%) | 11 (73.3%) | 81 (60.9%) | 33 (48.5%) |  | 18 (32.1%) | 0 (0%) | 16 (33.3%) | 15 (34.9%) |  |
| 24 months apart | 35 (41.7%) | 4 (26.7%) | 52 (39.1%) | 35 (51.5%) |  | 38 (67.9%) | 3 (100%) | 32 (66.7%) | 28 (65.1%) |  |
| **Wealth index** |  |  |  |  | **0.322** |  |  |  |  | **0.001** |
| High | 10 (11.9%) | 1 (6.7%) | 15 (11.3%) | 15 (22.1%) |  | 13 (23.2%) | 0 (0%) | 0 (0%) | 1 (2.3%) |  |
| Low | 37 (44.0%) | 6 (40.0%) | 61 (45.9%) | 31 (45.6%) |  | 20 (35.7%) | 1 (33.3%) | 19 (39.6%) | 16 (37.2%) |  |
| Middle | 37 (44.0%) | 8 (53.3%) | 57 (42.9%) | 22 (32.4%) |  | 23 (41.1%) | 2 (66.7%) | 29 (60.4%) | 26 (60.5%) |  |
